# Synthesis, Characterization and Quantification of Simvastatin Metabolites and Impurities

**DOI:** 10.3797/scipharm.1105-16

**Published:** 2011-07-25

**Authors:** Manish S. Bhatia, Swapnil D. Jadhav, Neela M. Bhatia, Prafulla B. Choudhari, Kundan B. Ingale

**Affiliations:** Department of Pharmaceutical Chemistry, Bharati Vidyapeeth College of Pharmacy, Near Chitranagari, Kolhapur-416013(M.S.), India

**Keywords:** Simvastatin, β-Hydroxy acid, Methyl ether of simvastatin, RP-HPLC

## Abstract

Simvastatin is used in treatment of hypercholesterolemia because it regulates cholesterol synthesis as a result of its β-hydroxy acid acting as an inhibitor of 3-hydroxy-methylglutaryl coenzyme A (HMG-CoA). The present communication deals with synthesis, characterization and development of accurate, precise and sensitive Reverse Phase High Performance Liquid Chromatography (RP-HPLC) method for simultaneous estimation of simvastatin and its synthetic impurities. The impurities methyl ether and β-hydroxy acid of simvastatin were synthesized in the laboratory and characterized by MS, NMR and FT-IR spectroscopy. The separation of simvastatin and its impurities was carried out on an isocratic JASCO RP-HPLC system using KYA TECH HIQ SIL C_18_ column (150 × 4.6 mm internal diameter, particle size 5 μm) operating at ambient temperature using acetonitrile:water (80:20 v/v) with 0.1% orthophosphoric acid as mobile phase. The method developed for HPLC analysis of three impurities along with simvastatin was validated using ICH Q2B (R1) guidelines and it complied with these guidelines. The results of analysis were found to be in the range of 98.14% to 101.89% for all analytes with acceptable accuracy and precision. The method can be used for detection and quantification of synthetic impurities in bulk or formulations of simvastatin.

## Introduction

Simvastatin, (1*S*,3*R*,7*S*,8*S*,8a*R*)-8-{2-[(2*R*,4*R*)-4-hydroxy-6-oxotetrahydro-2*H*-pyran-2-yl]-ethyl}-3,7-dimethyl-1,2,3,7,8,8a-hexahydronaphthalen-1-yl 2,2-dimethylbutanoate, is an anti-lipidemic prodrug which on in-vivo activation to its β-hydroxy acid (BHA) acts as an inhibitor of 3-hydroxy-3-methyl-glutaryl coenzyme A (HMG-CoA) reductase and hence regulates cholesterol synthesis. It is mainly used for the treatment of primary hypercholesterolemia, as it effectively reduces the levels of total and low-density lipoproteins (LDL), triglycerides and apolipoprotein B in plasma [1]. Simvastatin is obtained by synthesis from lovastatin (LOVA) by replacement of 2-methylbutyryl side chain with 2,2-dimethylbutyryl group [2].

Strict regulatory guidelines of the International Conference on Harmonization (ICH) have led to an increasing need for identification and quantification of trace impurities in drugs. The ICH defines impurities as any component of a pharmaceutical product which is not the chemical entity of active substance or excipients. Such impurities if present at levels higher than 0.1% need to be identified and qualified with appropriate toxicological studies. If impurities were expected to be very toxic then identification and qualification would be required even at lower concentrations [3].

Synthesis and characterization of impurities and clinically significant metabolites of new drugs utilizing optimum time and resources is one of the areas of current pharmaco-economic and clinical interest. Adequate separation, selectivity and sensitivity of detection and accurate quantification are always the prime concerns of pharmaceutical analyst in such work.

**Fig. 1.**
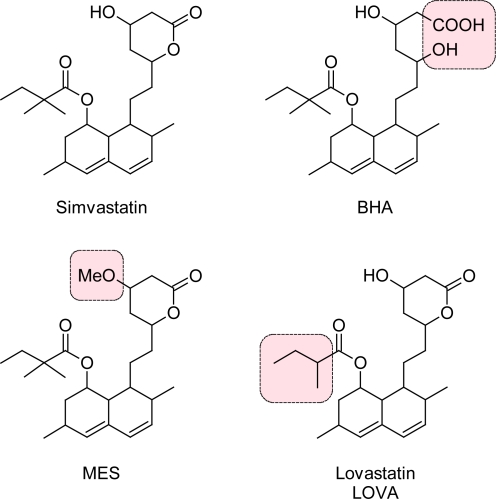
Structures of Simvastatin and Its impurities

A large number of impurities expected to be present in simvastatin were identified, quantified by several analytical techniques like HPLC-UV and LC-MS [4, 5]. Several methods for simultaneous estimation of simvastatin and its metabolite, BHA in biological fluids are published [6–10]. In the present communication, we have succeeded in synthesis and characterization of BHA and methyl ether of simvastatin (MES). This communication emphasizes use of non-compendial reference standards for quantification of analytes by chromatographic methods. The previously reported methods for quantification of impurities of simvastatin are having higher run time and employ various mobile phase modifiers like isopropyl alcohol [4]. A HPLC-UV method for simultaneous quantification of BHA, MES and LOVA along with simvastatin (Fig. 1) has been developed with improved analysis time and sensitivity of quantification than the previously reported methods [5].

## Result and Discussion

Various regulatory authorities like ICH, USFDA, Canadian Drug and Health Agency are emphasizing on the purity requirements and the identification of impurities in Active Pharmaceutical Ingredient’s (API’s). Qualification of the impurities is the process of acquiring and evaluating data that establishes biological safety of an individual impurity; thus, revealing the need and scope of impurity profiling of drugs in pharmaceutical research [11].

Identification of impurities is done by variety of chromatographic and spectroscopic techniques, either alone or in combination with other techniques. There are different methods for detecting and characterizing impurities like TLC, HPLC, HPTLC and AAS [12]. Conventional liquid chromatography, particularly, HPLC has been exploited widely in field of impurity profiling; the wide range of detectors, and stationary phases along with its sensitivity and cost effective separations have attributed to its varied applications.

Simvastatin is synthesized semi-synthetically from LOVA in a multistep reaction [2]. Simvastatin may contain various types of impurities as reported in European Pharmacopoeia as shown in Tab. 1 [13]. The most of simvastatin impurities are not toxic but considering purity and quality attributes of any drug in bulk, it is mandatory to perform impurity profiling of simvastatin.

In present work, three impurities of simvastatin were targeted. Synthesis and characterization of two impurities and analytical method development for simultaneous estimation of three impurities along with simvastatin was carried out.

The first impurity targeted was BHA which is active metabolite of simvastatin, formed by de-esterification of cyclic lactone in simvastatin. Simvastatin hydrolyses to BHA on absorption of moisture. As it is metabolite of simvastatin, it is said to be qualified and does not require toxicity profiling [3]. The second impurity targeted was LOVA which is starting material for simvastatin synthesis. The third impurity MES is a byproduct expected to be present due to use of methanol in one of steps of simvastatin synthesis.

**Fig. 2.**
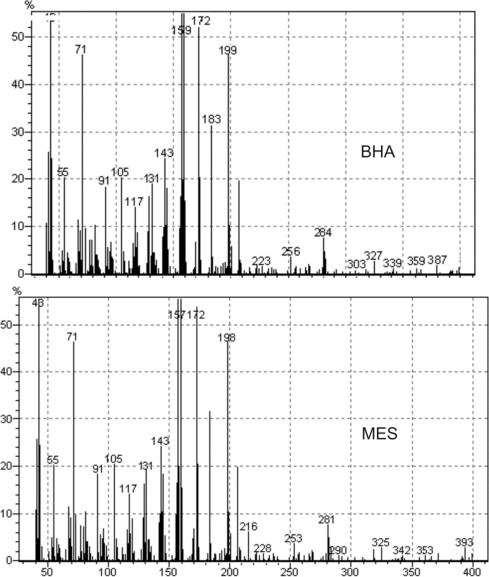
Mass Spectra of BHA and MES

**Fig. 3.**
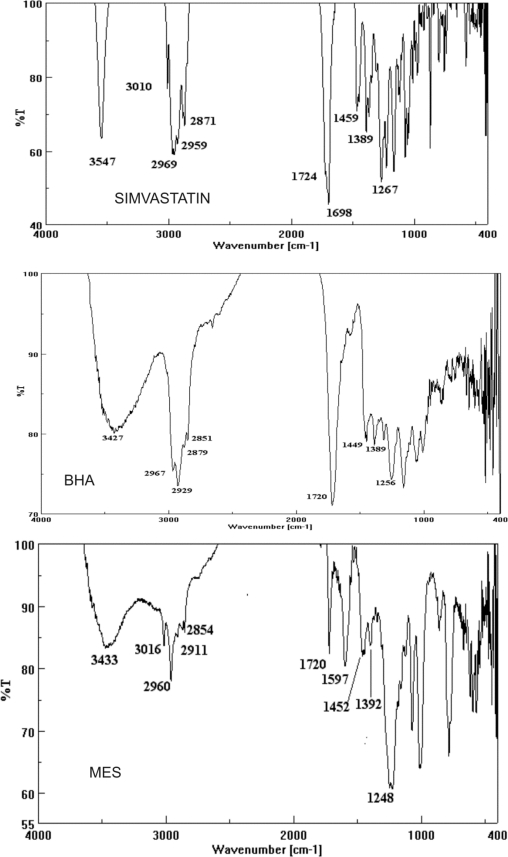
FT–IR Spectra of Simvastatin, BHA and MES

**Fig. 4.**
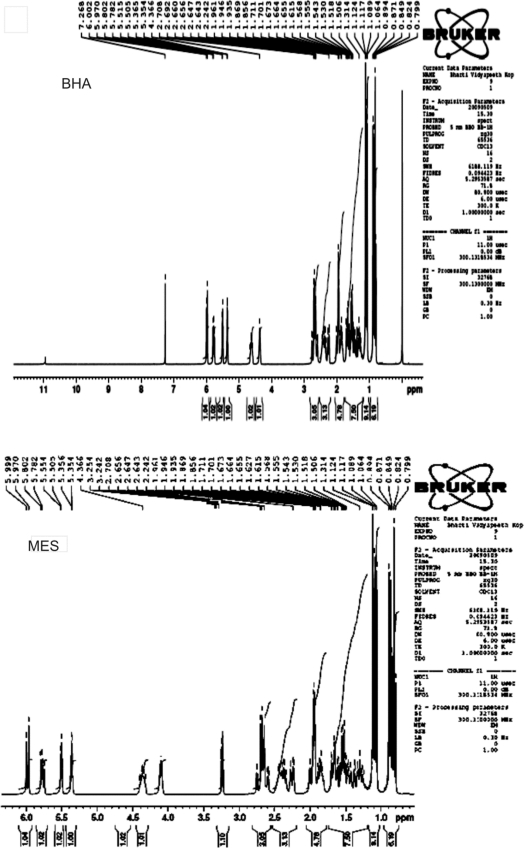
NMR Spectra of BHA and MES.

The chromatographic methods rely heavily on reference standard to provide accurate data. Reference standard needs to be a highly purified compound that is well characterized. Reference standards are of two types USP-NF and non-compendial reference standards. The USP-NF reference standards are official in USP, synthesized in reputed synthetic laboratories and are of high purity (nearly 99.99%). Their purity and cost is usually very high and thus do not require initial characterization before their use. Non-compendial reference standards are synthesized in any laboratory with reasonable efforts but require thorough characterization to assure its identity, strength, quality and purity so that they can be used in quantification of impurities [14]. Non-compendial reference standards are usually cheaper and can be used after thorough characterization. After thorough characterization we have successfully used laboratory synthesized BHA and MES as non-compendial reference standards for their quantification from Simvastatin samples by RP-HPLC. The BHA was synthesized by alkaline hydrolysis of simvastatin while MES has been synthesized using dimethyl sulfate for methylation of β-hydroxy group of lactone ring. These impurities have been characterized by spectroscopic techniques MS, FT-IR and NMR. The mass spectra, FT-IR spectra and NMR spectra are reported in Fig. 2, Fig. 3 and Fig. 4 respectively. The analysis of spectral data confirmed the synthesis of products with desired purity and quality. Hence, these impurities have been used as non-compendial reference standard in quantification of impurities of simvastatin.

The presently reported method is found to estimate three impurities along with simvastatin in run time of 10 minutes. In the development of this method, the first mobile phase tried was 50:50 acetonitrile: water (0.1% OPA) which elutes simvastatin at retention time of 41 min. In the next trial, 80:20 acetonitrile: water (0.1% OPA) was used as mobile phase which elutes simvastatin at 9 min and impurities were eluted before simvastatin at retention times of 5.478 (MES), 6.475 (BHA) and 7.388 (LOVA) min. This method can effectively estimate these impurities at concentration level of 0.1 % and less. The methods are validated by using ICH Q2R1 (Q2B) guidelines. Linearity has been studied by plotting two calibration curves, one with geometrically increasing concentrations and other with arithmetically increasing concentrations. The calibration curve with arithmetically increasing concentrations is required for quantification of analytes in sample solutions whereas the calibration curve with concentrations increasing in geometric progression serve the purpose of accessing the range over which the calibration curve could be used for quantification. The recovery studies of impurities were found to be in range of 98.21 to 100.72, proving accuracy of the developed method. Intra and inter day precision studies proves the repeatability and reproducibility of method. The analytical responses of impurities were found to be linear in concentration range of 0.2–25.6 μg/mL. The limits of detection and quantification of impurities were found to be lower than reported limit of impurities in European Pharmacopoeia as shown in Tab. 1.

The reported method is simple, precise, accurate and rapid for quantification of simvastatin impurities along with simvastatin in its bulk form or commercial formulations. As BHA is also a metabolite of simvastatin, further optimization of the method for quantification of BHA in biological fluids could make it useful for clinical and bioequivalence studies.

## Experimental

### Reagents and Chemicals

The pure drug simvastatin and LOVA were procured from Biocon Lab. Bangalore (India) as gift sample. All HPLC grade chemicals used were procured from S.D. Fine Chem., Mumbai. Water used for analysis was glass distilled using simple glass distillation assembly and filtered through 0.2 μm syringe filter. The impurities, BHA and MES were synthesized in laboratory.

### HPLC System

The separation of simvastatin and its impurities was carried out on an isocratic JASCO RP-HPLC system using KYA TECH HIQ SIL C_18_ column operating at ambient temperature (150 × 4.6 mm i.d., particle size 5 μm).The pump used in this HPLC system was PU 2080 pump (Dual piston with gear driven pump). The 20 μL sample solutions of analytes were injected to chromatographic system using Rheodyne Injector. The UV detector used in this HPLC system was Czerny turners mount monochromator with deuterium lamp as light source. The chromatographic and the integrated data were recorded using Hercule 2000 (interface) computer system. Data processing was carried out using Borwin ® Version 1.5 software. The HPLC analysis was carried out using acetonitrile: water (80:20 v/v) with 0.1% orthophosphoric acid (OPA) as a mobile phase at a flow rate of 1.0 mL/min in an isocratic elution mode. Before delivering the mobile phase in to the system, it was degassed using sonicater and filtered through 0.20 μm syringe filter. The detection was performed at 237 nm.

### Gas Chromatography-Mass Spectroscopy

A Simadzu QP 3010 mass spectroscopy interfaced with gas chromatography via electron impaction source was used for mass analysis and detection. A flow rate of 1 mL/min was used for sample analysis using Helium as mobile phase. The electron impaction source temperature was maintained at 280 nm. The detector used was gas chromatographic real analyzer. The column used was having 300 m length with 0.2 mm internal diameter.

### NMR Spectrometer

The NMR spectrometer used for analysis was of Brucker Company; model AVNCE-300 MHz. The NMR spectra of BHA and MES were recorded using CDCl_3_ as solvent.

### Synthesis of Impurities

**Fig. 5.**
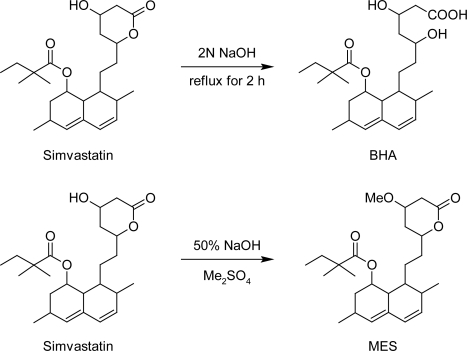
Synthesis of BHA and MES from Simvastatin

BHA: In 100 mL round bottom flask, 50 mL methanol and 50 mg simvastatin were taken. To this, 25 mL of 2N sodium hydroxide was added. It was then allowed to reflux for 2 h on water bath. It was then cooled and acidified with concentrated hydrochloric acid till solution was acidic to methyl orange. This solution was extracted 3 times with 10 mL of HPLC grade chloroform. Chloroform layer was separated. It was poured in petridish for evaporation. Solid, BHA was separated out. Product was then recrystallized with methanol (top, Fig. 5)

MES: In 50 mL round bottom flask, 1 g simvastatin and 10 mL of dichloromethane was taken. To this, 5 mL of 50 % sodium hydroxide was added. Solution was stirred vigorously for 20 min and cooled in ice- bath. Dimethyl sulfate solution was added drop wise till precipitation. The precipitate was washed with dilute ammonia solution to neutralize excess dimethyl sulfate. Precipitate was recrystallized with methanol. (bottom, Fig. 5).

LOVA: Pure drug procured from pharmaceutical company as gift sample was used as standard for LOVA which is also a synthetic impurity of simvastatin.

### Method Validation

The method was validated according to ICH Q2R1 (Q2B) guidelines, [15] meant for validation of analytical methods to check accuracy, precision, linearity range, limit of detection, limit of quantitation and robustness.

**Fig. 6.**
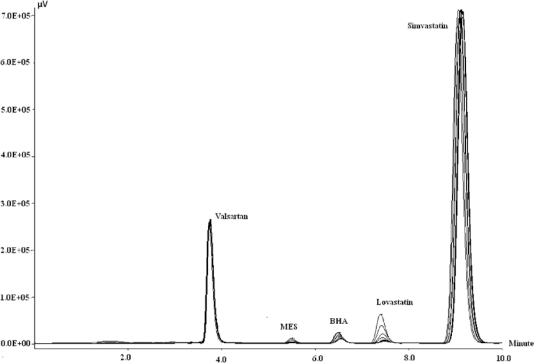
Overlain Chromatogram of Increasing Impurities Concentrations

**Fig. 7.**
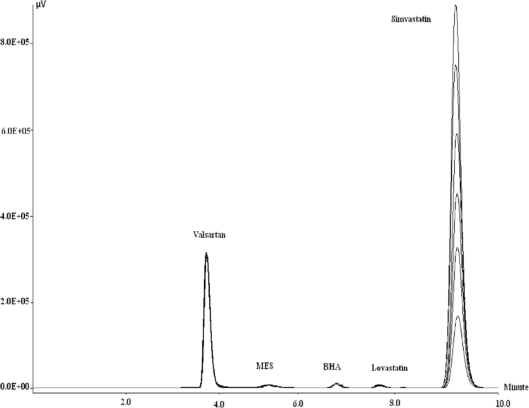
Overlain Chromatogram of Increasing Simvastatin Concentrations

Linearity Study: Standard working solutions of 10 μg/mL of simvastatin, LOVA, MES and BHA of simvastatin were prepared using mobile phase as a solvent. Required volume of solution from standard working solution was taken to get final dilutions of required strength for calibration curves and volume was made up with mobile phase. The HPLC analysis of all aliquots was carried out and response factor for each analyte was calculated. Two calibration curves were developed, one with geometrically increasing impurities concentrations (Fig. 6) and other with arithmetically increasing simvastatin concentrations (Fig. 7).The analytical responses of impurities were found to be linear in concentration range of 0.2 μg/mL to 25.6 μg/mL whereas analytical response of simvastatin was found to be linear in concentration range of 50 μg/ mL to 300 μg/mL. This study was carried out for three consecutive days. The laboratory samples were prepared using stock solutions of drug and impurities covering entire range of calibration curve. Amount of simvastatin and impurities present in laboratory sample were calculated using calibration curve data (Tab. 2) obtained by area normalization method. Results of laboratory sample assay are reported in Table 3.

Specificity: The specificity of the RP-HPLC method was determined by comparison of the chromatogram of mixed standards and individual analyte standard sample solutions. The parameters like retention time, resolution and tailing factor were calculated. Good correlation was found between the results of mixed standards and sample solutions.

Accuracy: Recovery studies were performed by standard addition method at three levels i.e., 80%, 100% and 120%. Known amounts of standard simvastatin and its impurities were added to pre-analyzed samples and they were subjected to analysis by the proposed HPLC method. Results of recovery studies are shown in Tab. 3.

Precision: Precision study was performed to find out intra-day and inter-day variations. The results of precision studies are reported in Tab. 4 and values of relative standard deviation less than 2% indicates high degree of precision.

System Suitability Parameters: These parameters were determined on freshly prepared standard stock solutions of simvastatin and its impurities. These analytes were injected into the chromatographic system under the optimized chromatographic conditions. Parameters that were studied to evaluate the suitability of the system are number of theoretical plates, tailing factor, resolution, separation factor etc [16] and are reported in Table 5.

Limit of detection (LOD) and limit of quantitation (LOQ); The LOD and LOQ were separately determined based on the calibration curve data. The standard deviation of the y-intercepts and slope of the regression lines were used in calculating these values using formulae give below.

$$\begin{array}{ll} \text{LOD }=\;3\text{.3}\times \frac{\sigma }{S} & \text{LOQ }=\;10 \end{array}\times \frac{\sigma }{S}$$

where, σ = standard deviation of the response and S = slope of the calibration curve

The LOD for MES, BHA, LOVA and simvastatin were found to be 0.030, 0.026, 0.031 and 0.045μg/mL respectively. The LOQ for MES, BHA, LOVA and simvastatin were found to be 0.094, 0.081, 0.91 and 0.135μg/mL respectively.

Robustness: The robustness study was carried out by making small changes in the optimized method parameters like ± 0.1 change in pH, ± 2% change in mobile phase component ratio and ± 0.1mL/min change in flow rate. These changes produced no significant impact on percentage recoveries of drugs. The results of the robustness study indicated that the developed method is robust and is unaffected by small variations in the chromatographic conditions.

## Figures and Tables

**Tab. 1.** European Pharmacopoeia Limits for Impurities in Simvastatin

**Name**	**% limit**
Simvastatin Hydroxy Acid	0.4 %
Epilovastatin and LOVA	1.0 %
Any other individual impurity	0.1 %
Total impurity other than LOVA and Epilovastatin	1.0 %

**Tab. 2.** Linearity Study of Simvastatin, LOVA, BHA and MES

**Parameters**	**Simvastatin**	**LOVA**	**BHA**	**MES**
Regression Equation	Y = A + B*C			
Range in μg/mL	50–300	0.2–25.6	0.2–25.6	0.2–25.6
Slope (B)	4.892 × 10^−1^	2.708 × 10^−3^	1.098 × 10^−3^	9.964 × 10^−3^
Intercept (A)	5.435 × 10^−1^	9.889 × 10^−2^	5.558 × 10^−2^	2.957 × 10^−2^
SE^a^ of Slope	1.248 × 10^−2^	7.547 × 10^−5^	9.647 × 10^−5^	5.197 × 10^−5^
SE^a^ of Intercept	2.347 × 10^−2^	1.457 × 10^−4^	2.912 × 10^−4^	0.107 × 10^−4^
Correlation coefficient (r)	0.9991	0.9993	0.9990	0.9992

C: Concentration in μg/ml Y: Unit of Response Factor a: Standard Error

**Tab. 3.** Results of Analysis and Recovery studies

**Analyte**	**Concentration in μg/mL**	**% Concentration Estimated^a^** **(Mean ± % R.S.D.^b^)**	**Amount Added in mg**	**% Recovery Estimated^a^** **(Mean ± %R.S.D.^b^)**

		99.91 ± 0.6705	80	98.37 ± 0.7846
Simvastatin	100	99.98 ± 1.2547	100	99.31 ± 0.3204
		100.51 ± 1.1886	120	99.37 ± 1.1101

		99.59 ± 0.7455	10	99.51 ± 0.2451
LOVA	12.5	99.45 ± 0.5277	12.5	99.12 ± 0.6289
		100.04 ± 0.3240	15	99.57 ± 0.3009

		100.74 ± 1.5565	10	99.42 ± 1.9385
BHA	12.5	99.24 ± 0.7042	12.5	99.88 ± 0.6470
		99.21 ± 1.0107	15	99.58 ± 0.8047

		100.39 ± 0.3642	10	99.59 ± 0.5019
MES	12.5	99.59 ± 0.27676	12.5	99.72 ± 0.5074
		100.42 ± 0.4497	15	99.87 ± 0.8337

a Average of Three Determinations;

b Relative Standard Deviation.

**Tab. 4.** Results of Precision Studies

**Parameter**		**% Concentration Estimated^a^** **(Mean ±% R.S.D.^b^)**			

**Analyte**		**Simvastatin**	**LOVA**	**BHA**	**MES**

**Repeatability**					

Laboratory Sample Analysis		99.32±1.7341	98.54±0.9547	98.33 ±1.1558	99.04 ± 1.2422

**Intermediate Precision**					

Day1	Morning	99.98±0.3854	96.04 ± 1.2422	98.36 ±0.6333	98.11 ± 0.6098
	Evening	101.11±0.6739	98.33 ± 1.1558	98.37 ±0.7874	99.60 ± 0.6613
Day2	Morning	101.02±0.9637	97.40 ± 1.2844	97.40 ±1.2844	97.98 ± 1.342
	Evening	99.84±1.6214	97.56± 1.4750	97.64 ±1.7892	96.52 ±1.2500

a Average of Nine Determinations;

b Relative Standard Deviation.

**Tab. 5.** System Suitability Parameters

**Parameter**	**Recommended Values**	**MES**	**BHA**	**LOVA**	**Simvastatin**
Capacity factor	> 2	2.947	3.0263	2.1894	2.0421
			2.86		
Resolution	> 2	3.54		4.57	
Retention Time in minutes	–	5.4	6.4	7.4	9.0
Tailing factor	≤2	1.2	1.2	1.26	1.21
Theoretical plates number	> 3000	6842.81	7083.81	7642.3	9044.12
